# Comparison of Three Ratiometric Temperature Readings from the Er^3+^ Upconversion Emission

**DOI:** 10.3390/nano10040627

**Published:** 2020-03-28

**Authors:** Aleksandar Ćirić, Jelena Aleksić, Tanja Barudžija, Željka Antić, Vesna Đorđević, Mina Medić, Jovana Periša, Ivana Zeković, Miodrag Mitrić, Miroslav D. Dramićanin

**Affiliations:** 1 Vinča Institute of Nuclear Sciences, University of Belgrade, P.O. Box 522, 11001 Belgrade, Serbia; aleksandarciric83@gmail.com (A.Ć.); tbarudzija@vinca.rs (T.B.); zeljkaa@gmail.com (Ž.A.); mina@vinca.rs (M.M.); jburojevic@vinca.rs (J.P.); zekovicivana@gmail.com (I.Z.); mmitric@vinca.rs (M.M.); 2 Faculty of Sciences and Mathematics, University of Niš, Višegradska 33, 18000 Niš, Serbia; jelena.aleksic@pmf.edu.rs

**Keywords:** luminescence thermometry, lanthanides, YF_3_, Er^3+^ emission, upconversion

## Abstract

The emission of Er^3+^ provides three combinations of emission bands suitable for ratiometric luminescence thermometry. Two combinations utilize ratios of visible emissions (^2^H_11/2_→^4^I_15/2_ at 523 nm/ ^4^S_3/2_→^4^I_15/2_ at 542 nm and ^4^F_7/2_→^4^I_15/2_ at 485 nm/ ^4^S_3/2_→^4^I_15/2_ at 545 nm), while emissions from the third combination are located in near-infrared, e.g., in the first biological window (^2^H_11/2_→^4^I_13/2_ at 793 nm/ ^4^S_3/2_→^4^I_13/2_ at 840 nm). Herein, we aimed to compare thermometric performances of these three different ratiometric readouts on account of their relative sensitivities, resolutions, and repeatability of measurements. For this aim, we prepared Yb^3+^,Er^3+^:YF_3_ nanopowders by oxide fluorination. The structure of the materials was confirmed by X-ray diffraction analysis and particle morphology was evaluated from FE-SEM measurements. Upconversion emission spectra were measured over the 293–473 K range upon excitation by 980 nm radiation. The obtained relative sensitivities on temperature for 523/542, 485/542, and 793/840 emission intensity ratios were 1.06 ± 0.02, 2.03 ± 0.23, and 0.98 ± 0.10%K^−1^ with temperature resolutions of 0.3, 0.7, and 1.8 K, respectively. The study showed that the higher relative temperature sensitivity does not necessarily lead to the more precise temperature measurement and better resolution, since it may be compromised by a larger uncertainty in measurement of low-intensity emission bands.

## 1. Introduction

Today, luminescence thermometry is considered to be a mature technology with many applications in diverse areas and environments, such as electrical and mechanical engineering, biomedicine, nanotechnology, and microfluidics (some of the application examples can be found in Reference [1] and references therein). Still, breakthrough advances related to it can be expected in the future, particularly in developments of novel luminescence thermometry probes and improvements of temperature readout schemes. Temperature readout methods from luminescence can be primarily classified according to the temporal nature of the measurement, as time-integrated (steady-state) or time-resolved (concerned with the emission decay and rise times). In the former type, the ratio of intensities of emissions, the so-called luminescence intensity ratio (LIR), is the most utilized method as it is simple, self-referencing, and ratiometric, and thus unaffected by fluctuations in excitation and detection systems. It can be used with many different types of luminescence probes and does not require expensive equipment with benefits such as fast response time and good temperature resolutions. The most commonly researched LIR with trivalent lanthanide ion (Ln^3+^) activated luminescence thermometry probes is one which utilizes the ratio of two emissions originating from the two thermalized excited levels whose magnitude can be formulated by the Boltzmann-type equation [2]:

(1) $$LIR=\frac{I_{H}}{I_{L}}=B\exp\left(-\frac{\Delta E}{kT}\right),$$

where *k* = 0.695 cm^−1^ K^−1^ is the Boltzmann constant, ΔE is an energy difference between excited levels, and H and L abbreviate energetically higher and lower excited levels of Ln^3+^ (thermalized levels), respectively. The pre-exponential factor *B* is usually considered as a constant to be determined from the fit of experimental data to Equation (1) or estimated from the Judd–Ofelt parameters, if available [3]. It depends on the degeneracy of the excited levels *g*, the spontaneous emission rates *A*, and emission energy of the transition *hν*:

(2) $$B=\frac{g_{H}A_{H}h\nu_{H}}{g_{L}A_{L}h\nu_{L}}$$

The thermalization of levels occurs when the energy gap permits the population of a higher-energy level solely by the thermal energy and when the nonradiative transition rates between the two levels surpass the radiative transition rates. Traditionally, emissions from the adjacent Ln^3+^ excited levels have been considered for the Boltzmann-type LIR temperature readings. However, this approach suffers from the limitation of its relative sensitivity:

(3) $$S_{R}\left[\%K^{-1}\right]=\left|\frac{1}{LIR}\frac{dLIR}{dT}\right|\times 100\%=\frac{\Delta E}{kT^{2}}\times 100\%$$

since it depends solely on the ΔE value which is, for the Ln^3+^ adjacent excited energy levels, largest in the case of Eu^3+ 5^D_1_ and ^5^D_0_ levels (ca. 1750 cm^−1^). Thus, the research has come to a conundrum: the lower the Δ*E* the better the thermalization, but at the cost of the loss of sensitivity [4]. Additionally, when choosing LIRs of the higher Δ*E* to achieve the higher S_R_, the population of the H level is low at low temperatures, with the consequence of low measurement resolution due to the high values of uncertainty in the measurements. One of the solutions to the above problem is an inclusion of the third, higher energy level, not too much separated from the traditionally used H level, to allow for thermalization (from now on the traditionally used H level is reabbreviated as M–mid), with the following logic; if L and M are thermalized, and M and H are thermalized, then the L and H fractional population will also follow the Boltzmann distribution and Equation (1) will remain valid. In other words, after the first thermalization from L to M, the electron at the M level can undergo the following three paths: radiative or nonradiative deactivation, or further thermalization to the H level. As the Δ*E_H-L_* = Δ*E_H-M_* + Δ*E_M-_*_L_, the result will be increased relative sensitivity for the given ion, and in some cases, thermalization with ≥1750 cm^−1^ can be achieved. However, caution is needed because H levels tend to have very low intensities, especially at lower temperatures, shifting the usable temperature range and reducing the overall temperature resolution, Δ*T*, a fact that has often been neglected in thermometric research of this type. Thus, both the relative sensitivity and the relative uncertainties of LIR should be simultaneously considered. So far, we have only been aware of few investigations of the kind: on Dy^3+^ ion (Δ*T* = 6.8 K) [3], Nd^3+^ (Δ*T* not reported) [5], and Er^3+^ (Δ*T* = 1 K) [6]. 

The first LIR study of Er^3+^ goes back a few decades to Berthou and Jorgensen [7], and since then Er^3+^ has become the most used lanthanide ion luminescence thermometry [1]. By using the ^2^H_11/2_ and ^4^S_3/2_ levels, separated by ca. 750 cm^−1^ [8], the maximum achievable relative sensitivity is 1080/*T*^2^. It was only until recently that this three-level strategy for increasing the sensitivity was investigated for Er^3+^ ion, by including the next higher level, ^4^F_7/2_.^4^ Numerous Er^3+^ doped materials, codoped with Yb^3+^, were investigated for luminescent thermometry [9,10,11] by the upconversion pumping mechanism. These upconverting materials have drawn significant attention due to the excitation in the NIR region of a biologically transparent window [12], allowing for numerous biological and medical applications (for example, in photothermal therapies of tumors) [13]. Ideally, the separation of energy levels would be low enough to provide for sufficient thermalization at biologically important temperatures, but high enough for the clear separation of levels in the spectrum. Ideally, this would be achievable by Δ*E* = 700 cm^−1^. Another advantage for in vivo temperature sensing would be if emissions of LIR levels are within the biologically transparent window as well, such as reported for Nd^3+^ [14]. To our knowledge, the same NIR-NIR readouts have not yet been reported for Er^3+^.

Upconverting Yb^3+^/Er^3+^ codoped materials do possess all the desired properties: (1) a trio of thermally coupled emissive levels (^4^S_3/2_, ^2^H_11/2_ and ^4^F_7/2_), (2) NIR excitation by widely available, cheap and powerful 980 nm laser, (3) emissions in the first biologically transparent window from thermally coupled levels ^4^S_3/2_ and ^2^H_11/2_ to the first excited level ^4^I_13/2_, and (4) energy gap between ^4^S_3/2_ and ^2^H_11/2_ close to the ideal 700 cm^−1^ for biologically relevant measurements. This paper presents a comparative analysis of performances of 3 LIR readouts by upconversion with 980 nm excitation: (1) from traditionally employed ^4^S_3/2_→^4^I_15/2_ and ^2^H_11/2_→^4^I_15/2_ emissions, (2) ^4^S_3/2_→^4^I_15/2_ and ^4^F_7/2_→^4^I_15/2_, and (3) with NIR emissions ^4^S_3/2_→^4^I_13/2_ and ^2^H_11/2_→^4^I_13/2,_ with the goal of obtaining temperature readout sensitivities and resolutions, and investigating measurement repeatability. The choice of YF_3_ as the host matrix was due to its low phonon energy of ~500 cm^−1^ [15], high chemical stability [16], and high iconicity [17], providing an excellent ground for highly efficient luminescence.

## 2. Materials and Methods 

For the preparation of Y_0.78_Yb_0.2_Er_0.02_F_3,_ the following chemicals were used: yttrium oxide (Y_2_O_3_, 99.99%), ytterbium oxide (Yb_2_O_3_, 99.99%), erbium oxide (Er_2_O_3_, 99.99%), and ammonium hydrogen difluoride (NH_4_HF_2_, 98.5%). All chemicals were purchased from Sigma−Aldrich. To synthesize Y_0.78_Yb_0.2_Er_0.02_F_3_, the appropriate amounts of commercial Y_2_O_3_, Yb_2_O_3_, and Er_2_O_3_ were mixed with NH_4_HF_2_ according to the overall reaction:

0.78Y_2_O_3_ + 0.2Yb_2_O_3_ + 0.02Er_2_O_3_ + 6NH_4_HF_2_ → 2Y_0.78_Yb_0.2_Er_0.02_F_3_ + 6NH_4_F + 3H_2_O

The obtained mixture was first thoroughly ground in an agate mortar to ensure the best homogeneity and then heated, first in the air at 170 °C for 20 h and then at 500 °C for 3 h in a slightly reducing atmosphere (Ar-10% H_2_).

Phase identification of the synthesized powder sample was done through X-ray diffraction (XRD) measurements on a Philips PW 1050 diffractometer that uses CuK_α_ radiation (*λ* = 1.54178 Å). XRD measurement conditions were: 2*θ* range 20−70° with a step of 0.05° and a counting time of 3 s. Mean crystallite size and microstrain were calculated by the XFIT program based on the fundamental parameters approach to X-ray diffraction line-profile fitting [18]. SEM measurements were done on a JEOL JSM-7600F scanning electron microscope. The powder was deposited on a graphite sample holder and coated with a Pt layer of 5 nm thickness using PECS Gatan 682. Photoluminescence measurements were carried out on pellets prepared by pressing the Y_0.78_Yb_0.2_Er_0.02_F_3_ powder under the load of 2000 kg/cm^2^. Photoluminescence upconversion emission spectra were measured using a Fluorolog-3 spectrofluorometer (model FL3-221, Horiba JobinYvon, emission slit set to 1 nm) over the 293–473 K temperature range in 20 K steps. Samples were excited by a 980 nm solid-state laser radiation (model MDLH 980 3W, the excitation power set to 150 mW) and emission was detected over the 450–870 nm spectral range by using a fiber-optic bundle.

## 3. Results and Discussion

**Figure 1:**
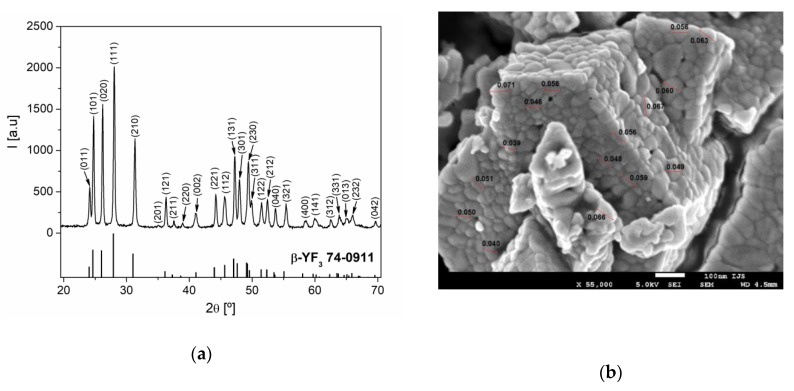
(**a**) The XRD pattern of Y_0.78_Yb_0.2_Er_0.02_F_3_ powder. Diffraction peaks are indexed according to PDF Card No. 74-0911; (**b**) SEM image of Y_0.78_Yb_0.2_Er_0.02_F_3_ sample.

The phase composition of the synthesized sample was examined by XRD analysis. As can be seen from Figure 1a, all the diffraction reflections can be indexed in a pure orthorhombic *β*-YF_3_ structure type, within the space group *Pnma* (62) [19]. From the XRD pattern, the calculated cell parameters were *a* = 6.3211 (4) Å, *b* = 6.8429 (4) Å, and *c* = 4.4162 (3) Å, and these values are in a good agreement with the values of cell parameters for YF_3_ from the work [19]. According to the analysis by the XFIT program, the mean crystallite size and microstrain of Y_0.78_Yb_0.2_Er_0.02_F_3_ sample were 51 nm and 0.2%, respectively.

The SEM image of the Y_0.78_Yb_0.2_Er_0.02_F_3_ sample is presented in Figure 1b. The examined sample consisted of strongly aggregated, irregularly shaped particles of approximately 20–100 nm in size.

**Figure 2:**
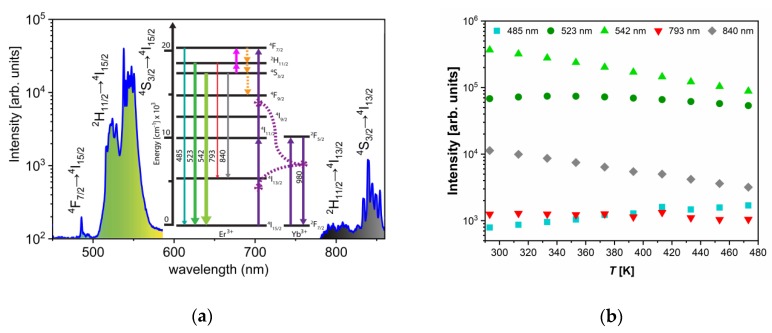
(**a**) Upconversion emission spectra of YF_3_:Yb^3+^/Er^3+^ excited by 980 nm (150 mW laser radiation) which show the excitation mechanisms and emission bands and electronic transitions of interest to luminescence thermometry; the thickness and color of emission arrows indicate the strength and color of upconversion emissions, respectively (the part of emission spectra between 600 and 750 nm where emission band due to ^4^F_9/2_ → ^4^I_15/2_ transition occurs is omitted since it is not of interest for this study); (**b**) temperature dependences of intensities of Er^3+^ upconversion emissions: 485 nm (turquoise square – ^4^F_7/2_→^4^I_15/2_ transition), 523 nm (dark green circle –^2^H_11/2_→^4^I_15/2_), 542 nm (light green up-triangle – ^4^S_3/2_→^4^I_15/2_), 793 nm (red down-triangle – ^2^H_11/2_→^4^I_13/2_), and 840 nm (gray diamond – ^4^S_3/2_→^4^I_13/2_).

Under the 980 nm excitation, YF_3_:Yb^3+^/Er^3+^ emissions were identified as those originating from Er^3+ 4^F_7/2_→^4^I_15/2_, ^2^H_11/2_→^4^I_15/2_, ^4^S_3/2_→^4^I_15/2_, ^2^H_11/2_→^4^I_15/2_, and ^4^S_3/2_→^4^I_15/2_ electronic transitions, centered at 485 nm, 523 nm, 542 nm, 793 nm, and 840 nm, respectively. The population of the high-energy levels of Er^3+^ is realized via well-known mechanisms: the absorption of 980 nm excitation by Yb^3+^ with a subsequent energy transfer to the Er^3+^, the ground state absorption of Er^3+^ (to a much less extent), and the excited state absorption of Er^3+^. The first energy transfer to the Er^3+^ ion is responsible for the population of the ^4^I_11/2_, while the second pumps from the ^4^I_11/2_ to the ^4^F_7/2_ (see Figure 2a). The population of the lower-lying ^2^H_11/2_ and ^4^S_3/2_ energy levels occurs via fast multiphonon de-excitations which compete with thermalization processes. These mechanisms allow for the efficient population of all three thermally coupled levels, ^4^F_7/2_, ^2^H_11/2_, and ^4^S_3/2_, with relative populations depending on the temperature in accordance to the Boltzmann distribution.

**Figure 3:**
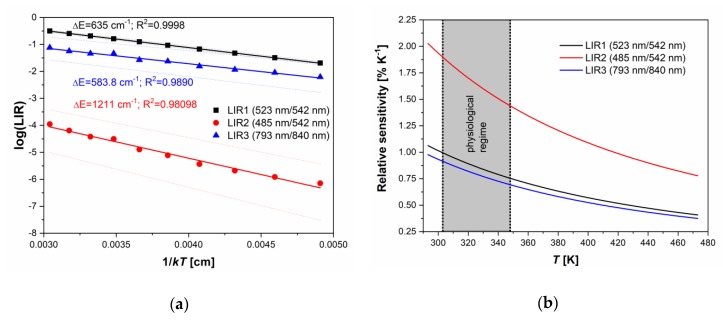
(**a**) The luminescence intensity ratio (LIR) dependence on 1/*kT*. Experimental data are given by symbols and the fits by full lines (black: LIR1 – the ratio of 523 and 542 nm emission intensities from ^2^H_11/2_ and ^4^S_3/2_ → ^4^I_15/2_ transitions; red: LIR2 – the ratio of 485 and 542 nm emission intensities from ^4^F_7/2_ and ^4^S_3/2_→^4^I_15/2_ transitions; blue: LIR3 – the ratio of 793 and 840 nm emission intensities from ^2^H_11/2_ and ^4^S_3/2_→^4^I_13/2_ transitions). The confidence intervals of fits are given by dashed lines, and the R^2^ is percentage of variation in the response that is explained by the linear regression model; (**b**) relative sensitivities of LIRs on temperature (black line – LIR1, red line – LIR2, and blue line – LIR3). At 293 K, relative sensitivity values, see Figure 3b, are 1.06 ± 0.02 (LIR1), 2.03 ± 0.23 (LIR2), and 0.98 ± 0.10%K^−1^ (LIR3). The LIR utilizing NIR upconversion emissions (LIR3) shows 0.87 ± 0.09%K^−1^ @310 K in the physiologically relevant range of temperatures (303–348 K range, which is relevant for biomedical applications of luminescence thermometry).

With the temperature increase, upconversion emissions from the ^4^S_3/2_ level (at 542 and 840 nm) rapidly decreased in intensity, while emissions from the ^2^H_11/2_ level (523 and 793 nm) showed almost constant intensities, Figure 2b. The intensity of emission from ^4^F_7/2_ showed a slight monotonic increase with temperature, Figure 2b, exactly like in the CaWO_4_:Yb^3+^/Er^3+^, as previously described by Li et al. [6]. The same phenomenon of the increase in the intensity of downshifting emission from the higher excited state has been described for Dy^3+^ activated CaWO4, where populations in Dy^3+ 4^G_11/2_ and ^4^I_15/2_ states enlarge with the temperature at the expense of the depopulation of the ^4^F_9/2_ state [4]. The enlarged population of ^4^F_7/2_ state occurs via thermalization from ^4^S_3/2_ level via ^2^H_11/2_ level, as described by Li et al. [6] showing that the ^4^F_7/2_ and ^4^S_3/2_ states are thermally linked with each other and that their populations follow Boltzmann’s law, Equation (1). This trend of changes in emission intensities with temperature provides an excellent ground for the study of performance of three different Boltzmann-type luminescence intensity ratio (LIR) based temperature readings available with Er^3+^. These are: LIR1—the conventional LIR which utilizes the ratio of two green (523 and 542 nm) upconversion emission intensities from ^2^H_11/2_ and ^4^S_3/2_ → ^4^I_15/2_ transitions; LIR2—which utilizes the ratio of blue and green (485 and 542 nm) upconversion emission intensities from ^4^F_7/2_ and ^4^S_3/2_→^4^I_15/2_, and where there is a larger energy difference between thermally coupled excited energy levels than in the conventional LIR1 (the energy of ^4^F_7/2_ level is higher than the energy of ^2^H_11/2_); and LIR3—which utilizes the ratio of two NIR upconversion emission intensities (793 and 840 nm) from ^2^H_11/2_ and ^4^S_3/2_→^4^I_13/2_ transitions and which utilizes the same pair of excited levels as conventional LIR1, see Figure 2a. The experimental values of these three LIRs are shown in Figure 3a (LIR1—black square symbols, LIR2—red circle symbols, and LIR3—blue triangle symbols) in the $\log\left(LIR\right)=-\Delta E\cdot \frac{1}{kT}+log\left(B\right)$ format that is obtained by taking the natural logarithm of Equation. (1). The log(LIR) vs $\frac{1}{kT}$ is a linear function with a slope of −ΔE which is the energy difference between thermalized excited levels, which is obtained by fitting of experimental data represented as full lines in Figure 3a (the 95% confidence intervals are given by dashed lines; the fitting parameters are given in Table 1).

LIR1 and LIR3 showed identical sensitivity to temperature (the difference being within the experimental error), see Table 1, which is expected since both LIRs are based on the same couple of thermalized energy levels (^2^H_11/2_ and ^4^S_3/2_). The energy difference between these levels of about 600 cm^−1^ obtained from the LIRs’ temperature dependence (ΔE) was slightly lower than one obtained from the spectral measurements, which is a common case in luminescence thermometry [20].

However, NIR upconversion emissions used for LIR3 were of much smaller intensities than green emissions used for LIR1, so that the uncertainty in the measurements of LIR3 was much larger than for that of LIR1, see Table 2.

For this reason, the temperature resolution of LIR3 of $\Delta T_{LIR3}=1.8K$ (at 313 K) was much worse than that of LIR1 $\Delta T_{LIR1}=0.3K$ ($\Delta T=\sigma_{R}/S_{R}$; $\sigma_{R}$ – the relative standard deviation of measurement). LIR2 has two times larger sensitivity (2.03%K^−1^ at 293 K) than LIR1 and LIR3, which is due to the two times larger energy difference between thermalized excited energy levels. This result confirms the assumption that relative sensitivities beyond the currently accepted limit for thermometry with Ln^3+^ ions may be achieved if LIR utilizes the emission from the excited level of higher energy than one used in the traditional practice of Boltzmann-type LIR. Nevertheless, as in the case of LIR3, the emission from the high energy excited levels usually has a weak intensity, so the uncertainty in measurement may be large. Here, the temperature resolution obtained with LIR2 was $\Delta T_{LIR2}=0.7K$, two and a half times worse than with LIR1, meaning that the larger relative sensitivity of the LIR does not necessarily lead to a more precise measurement. On the other hand, from the trend in emission intensity changes with temperature shown in Figure 2b, one may conclude that the use of emission from ^4^F_7/2_ transition (LIR2) may increase in importance at higher temperatures than measured in this research, where its intensity may be comparable with the emission intensity of ^2^H_11/2_. For such measurements, the luminescence probe ought to be temperature stable above 480 K. 

**Figure 4:**
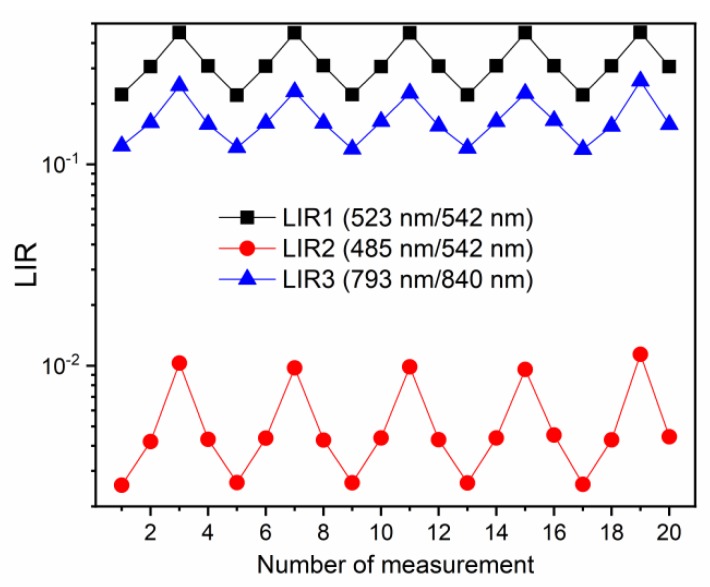
Repeatability of measurement tests of different LIRs (LIR1—black square symbols; LIR2—red circle symbols, LIR3—blue triangle symbols). The small variations in LIR values are within the value of uncertainty in measurements. The repeatability measurements were conducted at 313 K, 353 K, and 413 K.

For the investigation of repeatability of measurements, LIR values were evaluated for 20 measurements, at 313 K, 353 K, and 413 K, in the heating and cooling sequences, and the results are depicted in Figure 4 (LIR1—black square symbols; LIR2—red circle symbols, LIR3—blue triangle symbols). The repeatability of measurements was excellent with all three LIRs; the observed small variations in LIR values were on the level of uncertainty in measurements. Additionally, LIRs were unaffected by the heating/cooling, proving the temperature stability of the probe material in the used temperature measurement range. Such excellent results of repeatability testing are common in luminescence thermometry with Ln^3+^ probes [1].

It is important to note that this study has two limitations. Firstly, the power dependences of upconversion emission intensities on LIRs were not studied in detail (note that similar dependences on excitation power were found for all emission bands). Secondly, the conditions for obtaining the Boltzmann-based equilibrium between two excited levels were not checked (the nonradiative transition rates between the two levels should surpass the radiative transition rates within the considered temperature range) [21]. These analyses, although important, were beyond the main aim of the study which was to show three different types of LIRs available with Er^3+^ activated luminescence thermometry probes, and to compare their performance. Finally, one should be aware that continued exposure to excitation at 980 nm causes overheating in biological tissues due to a strong optical absorption of water and biological specimens [22]. The problem may be overcome by using the 915 nm excitation as shown in Reference [22] or by using Nd^3+^ as a codopant which renders the system excitable at 800 nm [23,24]. However, in the latter case, the Er^3+^ NIR LIR would be very difficult to measure.

## 4. Conclusions

Er^3+^ emission affords three combinations of luminescence intensity ratios suitable for the luminescence thermometry. The well-known LIR1, which utilizes two green emissions (centered at 523 and 542 nm), offers temperature measurements with the relative sensitivity of 1.06 ± 0.02%K^−1^ at 293 K and temperature resolution of 0.3 K when using 980 nm excited upconversion emission of Yb^3+^,Er^3+^:YF_3_ nanoparticles. Secondly, LIR3, which exploits NIR emissions (793 nm and 840 nm), shows a similar relative sensitivity, as expected since NIR emissions originate from the same excited levels as green emissions. This NIR LIR3 is suitable for biothermal applications because both excitation and emissions are within the biological transparency window. At 310 K, the NIR LIR3 offers 0.87 ± 0.09%K^−1^ relative sensitivity. However, smaller intensities of NIR emissions compared to green ones led to a higher value of uncertainty in measurement, so that the temperature resolution was only 1.8 K. The LIR2, which utilizes blue and green emissions (centered at 485 and 542 nm), e.g., emissions that originate from ^4^F_7/2_ and ^4^S_3/2_ energy levels, has two times higher sensitivity (2.03 ± 0.23%K^−1^) than the traditional LIR1 of green emissions. The higher relative sensitivity value is because of the larger energy difference between ^4^F_7/2_ and ^4^S_3/2_ levels than between ^2^H_11/2_ and ^4^S_3/2_. This result confirms the assumptions that the sensitivity limitations of Ln^3+^ LIRs may be overcome using emissions from high-energy excited levels. Still, the higher relative sensitivity of blue/green LIR2 compared to green/green LIR1 does not afford more precise temperature measurement since the achieved temperature resolution (0.7 K and 0.3 K, respectively) favors the traditional LIR1. The blue/green LIR2 may have good potential for use at temperatures higher than in this study, since the intensity of ^4^F_7/2_ blue emission constantly gains in intensity with an increase in temperature. Finally, one should note that temperature resolutions may be better if the measurements of emission spectra are improved over those used in this study. The Yb^3+^,Er^3+^:YF_3_ nanoparticles proved themselves as more than suitable for LIR based upconversion luminescence thermometry; the only limitation was the upper-temperature limit of 480 K, beyond which the upconversion emission intensity irreversibly lessens.

## Figures and Tables

**Table 1:** The fitting parameters of experimental luminescence intensity ratio (LIR) data from Figure 2 to the $\log\left(LIR\right)=-\Delta E\cdot \frac{1}{kT}+\log\left(B\right)$ function. Values of relative sensitivities, $S_{R}$, are calculated using Equation (3).

LIR	Involved Er^3+^ Transitions	$\Delta E\;[{\mathrm{cm}}^{-1}]$	log(B)	$S_{R}\;[\%K^{-1}]\;\mathrm{at}\;293.15\;K$
LIR1	H211/2→I415/2(523nm)S43/2→I415/2(542nm)	635.0	1.425	1.06 ± 0.02
LIR2	F47/2→I415/2(485nm)S43/2→I415/2(542nm)	1211.0	−0.372	2.03 ± 0.23
LIR3	H211/2→I413/2(793nm)S43/2→I413/2(840nm)	583.8	0.623	0.98 ± 0.10

**Table 2:** Uncertainties in LIRs at different temperatures; σ —the standard deviation of measurement, $\sigma_{R}$ —the relative standard deviation of measurement.

	LIR1	LIR2	LIR3
	313 K		
σ	0.000687	0.000034	0.001884
$\sigma_{R}$ [%]	0.3102	1.3043	1.5618
	353 K		
σ	0.001255	0.000094	0.003444
$\sigma_{R}$ [%]	0.4082	2.1678	2.1535
	413 K		
σ	0.001242	0.000729	0.015068
$\sigma_{R}$ [%]	0.2755	7.1597	6.3538
